# ESR Essentials: role of PET/CT in neuroendocrine tumors—practice recommendations by the European Society for Hybrid, Molecular and Translational Imaging

**DOI:** 10.1007/s00330-024-11095-7

**Published:** 2024-10-10

**Authors:** Ricarda Ebner, Gabriel T. Sheikh, Matthias Brendel, Jens Ricke, Clemens C. Cyran

**Affiliations:** 1 Department of Radiology, LMU University Hospital, LMU Munich, Munich, Germany; 2 Department of Nuclear Medicine, LMU University Hospital, LMU Munich, Munich, Germany

**Keywords:** Neuroendocrine tumors, Positron emission tomography computed tomography, Receptors (Somatostatin), Radiopharmaceuticals, Molecular imaging

## Abstract

**Abstract:**

Neuroendocrine neoplasms (NEN) originate from the secretory cells of the neuroendocrine system, with the majority arising in the gastrointestinal tract and pancreas. Given the heterogeneity in the biological behavior and morphological differentiation of these tumors, advanced imaging techniques are crucial for supporting the suspected diagnosis, accurate staging, and monitoring therapy. As most well-differentiated NEN demonstrate overexpression of somatostatin receptors (SSR) on the cell surface, SSR-directed PET/CT is considered the reference standard for imaging of this particular entity. SSR-PET/CT should be the imaging method of choice in every NEN G1 or G2 and considered for re-staging after both potentially curative and non-curative surgeries. The extent of SSR expression is also crucial for determining a patient’s eligibility for peptide receptor radionuclide therapy (PRRT). PRRT utilizes [^177^Lu]Lu-DOTA-TATE to target the SSR receptor and can significantly prolong progression-free survival in patients with advanced, progressive neuroendocrine tumor of the gastroenteropancreatic system (GEP-NET). PET/CT is a central component of the multidisciplinary management of NEN. Variable follow-up intervals are recommended, considering that tumors with higher proliferation rates or advanced metastatic disease require more frequent assessments. The combination with other imaging modalities, like MRI, complements SSR-PET/CT, further enhancing overall diagnostic accuracy.

**Key Points:**

- *Somatostatin receptor-PET/CT (SSR-PET/CT) is the guideline-recommended reference standard for imaging well-differentiated neuroendocrine tumors (NET)*.
- *SSR-PET/CT should be the diagnostic imaging of choice for staging and post-therapy re-staging of grade 1 or 2 NET (G1 or G2)*.
- *Variable follow-up intervals are recommended for NET G1 and G2. Tumors with higher proliferation rates or advanced metastatic disease necessitate more frequent assessments*.

## Key recommendations

- Optimal imaging and treatment planning: For supporting the suspected diagnosis, accurate staging, and therapy monitoring of neuroendocrine neoplasms, SSR-PET/CT with DOTA-conjugated somatostatin analogs should be utilized, especially due to its reference standard status for imaging well-differentiated NET and its role in determining eligibility for peptide receptor radionuclide therapy (PRRT) in advanced inoperable tumors with significant receptor overexpression (level of evidence: high).
- SSR-PET/CT for initial and re-staging: SSR-PET/CT should be the imaging method of choice in every NET G1 or G2, with few exceptions, and should be considered for re-staging after both potentially curative and non-curative surgeries, as it is crucial in complementing conventional imaging methods and ensuring comprehensive diagnosis and treatment planning (level of evidence: high).
- Follow-up interval: Recommended follow-up intervals (CT, MRI, or PET/CT) are 6–12 months for patients with NET G1 and NET G2 after complete resection or for those with residual tumors and a low Ki-67 index (< 5%), depending on the primary tumor site. For tumors with a high proliferation rate in NET G2 (Ki-67 > 5%) and neuroendocrine carcinomas (G3), more frequent follow-up examinations every 2 to 3 months are appropriate. Similar intervals apply to advanced metastatic disease (level of evidence: high).

## Introduction

Neuroendocrine neoplasms (NEN) are a rare and heterogeneous group of malignancies that arise from the secretory cells of the diffuse neuroendocrine system. The majority of NEN (~ 75%) develop in the gastrointestinal tract and pancreas, collectively referred to as gastroenteropancreatic NEN (GEP-NEN). The most common sites in the gastrointestinal tract include the small intestine (30.8%), rectum (26.3%), colon (17.6%), pancreas (12.1%), and appendix (5.7%). Another common site for the occurrence of NEN is the lungs [1].

For clinical purposes, NEN are categorized based on the morphological differentiation and biological behavior of the tumors. Well-differentiated neuroendocrine tumors (NET) are predominantly slow-growing and are stratified into three grades (G1-3) based on their Ki-67 expression; neuroendocrine carcinomas (NEC) are high-grade, poorly differentiated, aggressively growing tumors with a very high tumor cell proliferation rate. Appropriate imaging and treatment planning for NEN largely depend on the histopathological assessment of the proliferation rate [2].

Imaging is crucial for staging at primary diagnosis and follow-up of NEN. Although NET tends to progress slowly, regular follow-up imaging is essential. A multimodal diagnostic approach that combines anatomical-morphological and functional imaging allows for the early detection of subtle changes, ensuring timely intervention and optimal management.

In this article, we evaluate the importance and role of hybrid imaging with Positron emission tomography/ computer tomography (PET/CT) in patients with NEN, its potential applications, and potentially useful combinations of PET/CT with other imaging modalities.

## Imaging in neuroendocrine neoplasms

Nuclear medicine imaging of NEN is based on the overexpression of somatostatin receptors (SSR) on the cell surface of well-differentiated NET. Radiolabeled somatostatin analogs (SSA) bind to SSR subtypes 2 and 5 on the NET cell surface. Unfortunately, SSR-directed imaging is mostly not ideal for assessing poorly differentiated, aggressive NEC, as this tumor entity frequently lacks SSR overexpression. In such cases, PET imaging with Fluorine-18 [^18^F]fluorodeoxyglucose (FDG) is the better choice, depicting the increased glucose metabolism of NEC.

**Fig. 1:**
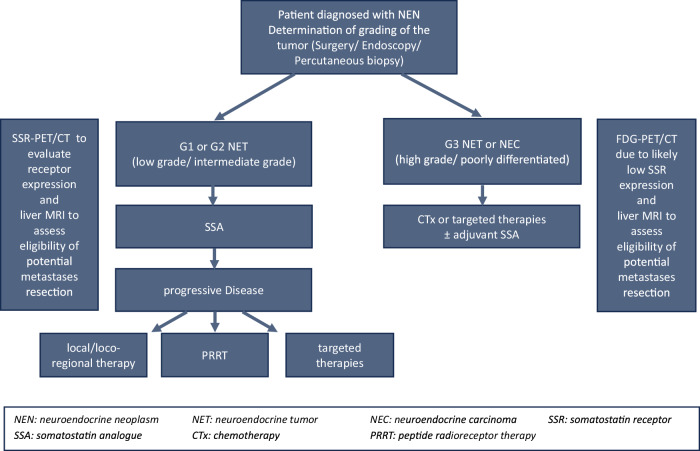
Diagnostic approach of patients diagnosed with NEN

PET/CT with somatostatin receptor analogs is the guideline-recommended standard of care in NET imaging. According to the 2023 guidance paper from the European Neuroendocrine Tumor Society (ENETS), SSR-PET/CT imaging is crucial for detecting both primary and metastatic sites in NET. The availability of SSR radiotracers varies significantly across regions. Gallium-68 [^68^Ga] based tracers, such as [^68^Ga]Ga-DOTA-TATE, [^68^Ga]Ga-DOTA-NOC, and [^68^Ga]Ga-DOTA-TOC, are predominantly utilized given their proven capability in imaging NET. Emerging [^18^F] labeled SSR tracers, including [^18^F]-SiFAlin-TATE, exhibit considerable potential, however, their commercial availability remains limited. Indium-111 [^111^In]In-DTPA-Octreotide scintigraphy or Technetium-99m [^99m^Tc]Tc-Tektrotyd single-photon emission computed tomography (SPECT)/CT can be used as alternative imaging modalities [3]. Planar SSR scintigraphy or SPECT/CT is recommended as an alternative only if PET/CT is not available, due to its lower spatial resolution, reduced diagnostic accuracy, higher radiation dose, and significantly longer procedure time. SSR imaging is also important for assessing a patient’s eligibility for peptide receptor radionuclide therapy (PRRT) in advanced inoperable tumors, where sufficient SSR expression is key. The prospective NETTER-1 study demonstrated the effectiveness of Lutetium-177 [^177^Lu]Lu-DOTA-TATE, which led to a significant extension in progression-free survival compared to high-dose octreotide long-acting release in patients with advanced, progressive midgut NET [4]. Figure 1 illustrates the diagnostic approach for patients diagnosed with NEN.

## Functional imaging

The integration of PET and contrast-enhanced CT (ceCT) marks a significant advance in the diagnosis of well-differentiated NET. PET provides additional functional information to established morphological imaging, facilitating the detection of the primary tumor and small metastases, while also significantly influencing therapeutic decisions [5].

Conventional CT plays a pivotal role in the diagnosis and management of NEN due to its widespread availability, high-resolution detail, and rapid examination speed, even for multiphasic protocols. It can assess tumor extension by showing whether the tumor is locally limited or has spread to surrounding structures, such as lymph nodes, visceral organs, or bones. Particularly for surgical planning, accurate anatomical knowledge of the tumor and its metastases is essential.

**Fig. 2:**
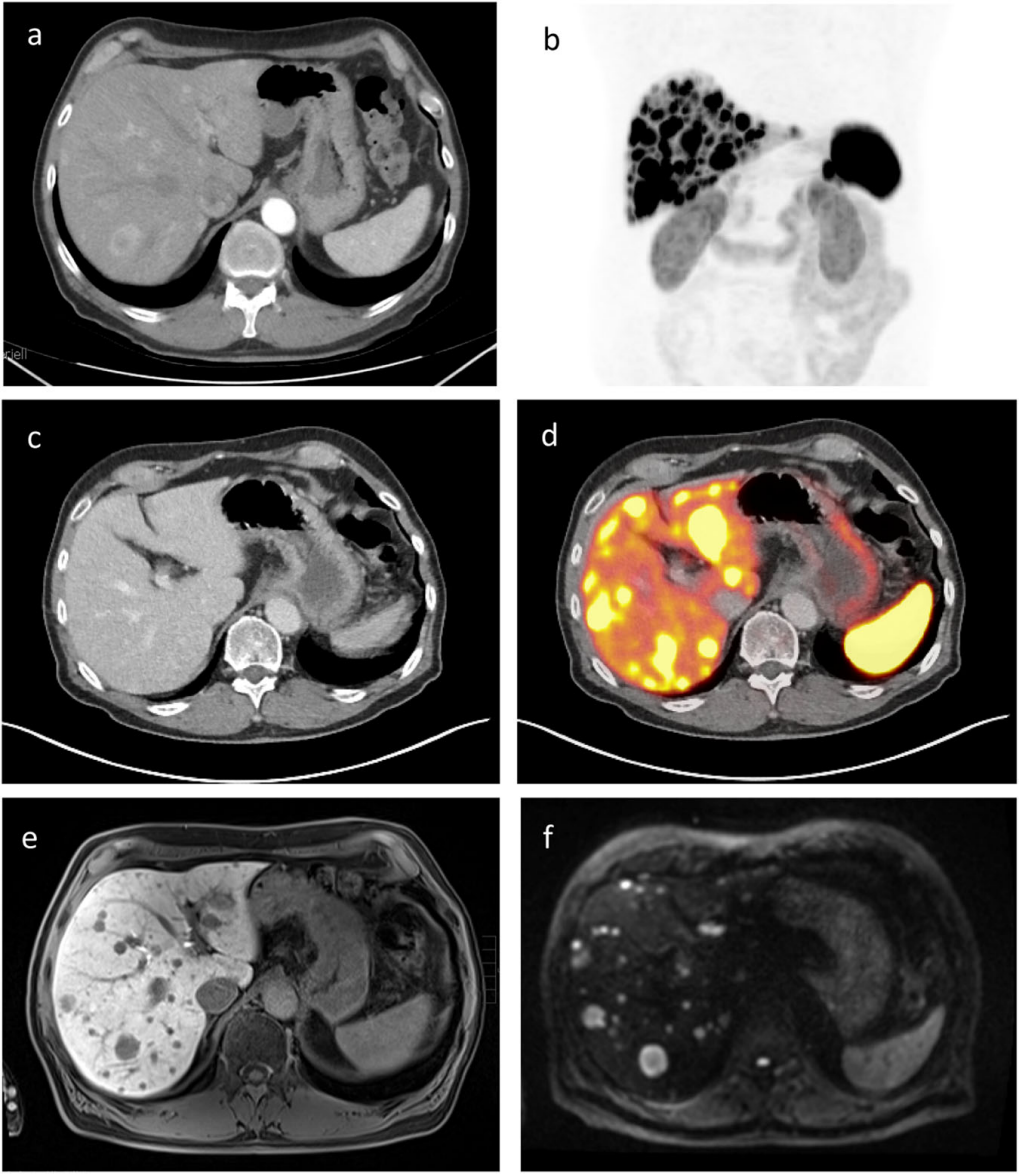
Patient presented with disseminated liver metastases (**b**) of histologically confirmed neuroendocrine tumor (G2, Ki-67 5%). Disseminated liver metastases exhibited early arterial contrast enhancement in ceCT (**a**) with subsequent early washout in portal venous phase (**c**). Liver metastases were clearly visible on magnetic resonance imaging (MRI) (including contrast-enhanced T1 sequence and diffusion-weighted imaging (DWI)-sequences) (**e**, **f**) and demonstrated intense SSR overexpression (**d**)

Patient preparation and a dedicated CT protocol have a major influence on the value of the examination and should therefore be carried out carefully and tailored to the respective diagnostic question. The use of an iodine-based intravenous contrast agent is important, as it significantly increases sensitivity in detecting tumors and metastases. For gastrointestinal NET, which typically shows significant arterial contrast uptake and tends to metastasize to the liver, multiphase CT scans of the upper abdomen are useful [6]. Figure 2 includes comprehensive imaging findings of a patient with a neuroendocrine tumor and disseminated liver metastases, highlighting their behavior across multiple diagnostic modalities.

Still, conventional CT alone has lower accuracy in identifying lymph node metastases compared with SSR-PET/CT [7]. One reason for this is the size threshold used in anatomical-morphological imaging to differentiate between benign and malignant lymph nodes. Smaller tumor lesions may therefore be missed or mistakenly classified as benign. Only about 65% of lymph node metastases detectable by SSR-PET/CT through radionuclide uptake are identifiable and accurately characterized using conventional imaging techniques. Also, the average accuracy of CT in detecting the primary tumor, liver metastases, and extrahepatic metastases is only 73%, 80%, and 75%, respectively [8].

Although an unenhanced low-dose scan may suffice for anatomical orientation and attenuation correction in PET/CT, a contrast-enhanced scan is recommended for a comprehensive evaluation of mediastinal and abdominal structures. The use of positive oral (iodine-containing) contrast agents is not recommended in CT scans to avoid missing small tumors within the intestinal and gastric walls [9].

### SSR-PET/CT

In clinical practice, a range of radiopharmaceuticals is available for SSR-directed imaging, each demonstrating a high affinity particularly for SSR subtype 2. The most frequently utilized SSA, including DOTA-TATE, DOTA-TOC, and DOTA-NOC, vary in their affinity for different SSR subtypes. However, linked with the radionuclide [^68^Ga], they are regarded as equally effective in terms of diagnostic accuracy for imaging NET [10].

SSR-PET/CT with [^68^Ga]-DOTA-conjugated SSA has been approved by the Food and Drug Administration and European Medicines Agency for imaging of well-differentiated NET and is now considered the ENETS-recommended reference standard for this purpose [8]. While the synthesis of [^68^Ga]-DOTA-conjugated peptides for NET imaging is well-established, reliable, and features relatively simple radiochemistry, challenges such as small-scale production and the short 68-min half-life of [^68^Ga] persist. However, the recent development and clinical application of a new SSR-ligand, [^18^F]-SiFAlin-TATE, offers significant improvements in acquiring SSR-PET/CT images for patients with NET. The cyclotron-produced radionuclide [^18^F] has a half-life of 110 min, providing an optimal window for synthesis, transport, and in-vivo distribution, while minimizing radiation exposure to the patient [8]. Furthermore, [^18^F]-labeled tracers emit positrons with lower energy, leading to higher-resolution images. For [^18^F]-SiFAlin-TATE, significantly higher tumor uptake was described in almost all tumor lesions in common metastatic sites of NET, including the liver, lymph nodes, and bone, but not in lung lesions [11]. The development of cyclotron-derived, [^18^F]-labeled compounds for NET imaging might solve the disadvantages of a cost-intensive Germanium-68 [^68^Ge]-/[^68^Ga]-generator, low activity amounts after single elution, and the shorter half-life of [^68^Ga]-labeled compounds [12].

Baseline SSR imaging with PET/CT is recommended by the ENETS guidelines for every NET G1 or G2 [13]. For G1-NET or G2-NET, SSR-PET/CT is recommended, except in specific cases such as gastric NET type I or G1 rectal NET with a diameter of less than 1 cm and no adverse features. In these instances, alternative diagnostic approaches (CT, MRI, ultrasound) may be more appropriate due to the typically indolent nature of these tumors and their lower probability of developing metastases. SSR imaging should be considered for re-staging after potentially curative surgery in patients with a clinically significant risk of residual or metastatic disease, even if SSR imaging was not performed before surgery. Similarly, SSR imaging is deemed necessary for re-staging after non-curative surgery in all patients, complementing conventional imaging methods [14]. When clinical or laboratory indicators suggest progression, the recommended imaging approach for SSR-positive NET involves combining SSR imaging with CT and/or MRI. If SSR imaging detects a new lesion on a PET scan while the anatomical-morphological CT scan shows stable disease, the disease may be indeed advancing. Some organs, such as the thyroid and adrenal gland, or inflammatory conditions, can accumulate SSR radiotracers due to their slight expression of SSR. This underscores the need for careful evaluation when interpreting scan results, especially because the CT component of SSR-PET/CT can significantly aid in diagnostic guidance [15].

In the evaluation of SSR-PET/CT scans, the description of malignant NET lesions, and their increased receptor density is traditionally documented in a descriptive manner. This assessment primarily relies on comparing the SSR-uptake of the NET manifestations to that of the liver, known as the Krenning score [16]. Recently, a new standardized reporting system, SSTR-RADS 1.0 has been introduced. This system is modeled after the established and widely used “Reporting and Data System” (RADS) frameworks that are widespread in other imaging modalities and tumor entities. SSTR-RADS 1.0 is specifically designed to standardize the interpretation of SSR-PET/CT scans and to aid in the decision whether or not NET patients are suitable for PRRT [17], a receptor-targeted radiotherapy for NET, aimed with high affinity and specificity at SSR and involves the systemic administration of a radioactively labeled peptide. DOTA-TATE combined with therapeutic beta-emitting radionuclides like [^177^Lu] or Yttrium-90 ([^90^Y]) enables effective treatment. PRRT is used in the management of inoperable or metastasized, well-differentiated NET and is particularly useful for patients with tumor progression under biotherapy with SSA. SSA play a pivotal role in managing NET, particularly in alleviating symptoms associated with carcinoid syndrome and tumor growth. However, for cases unsuitable for chemotherapy due to their slow growth behavior, such as NET G1 and G2, and when curative tumor resection or other local ablative methods are not feasible, PRRT emerges as a promising therapeutic option [18]. As de-differentiated, SSR-negative lesions cannot be targeted by PRRT, an additional [^18^F]FDG-PET/CT may be beneficial for patients with NET G2 or G3. This helps identify discordant FDG-positive, SSR-negative lesions, which are known prognostic markers for poor outcome after PRRT [19].

**Fig. 3:**
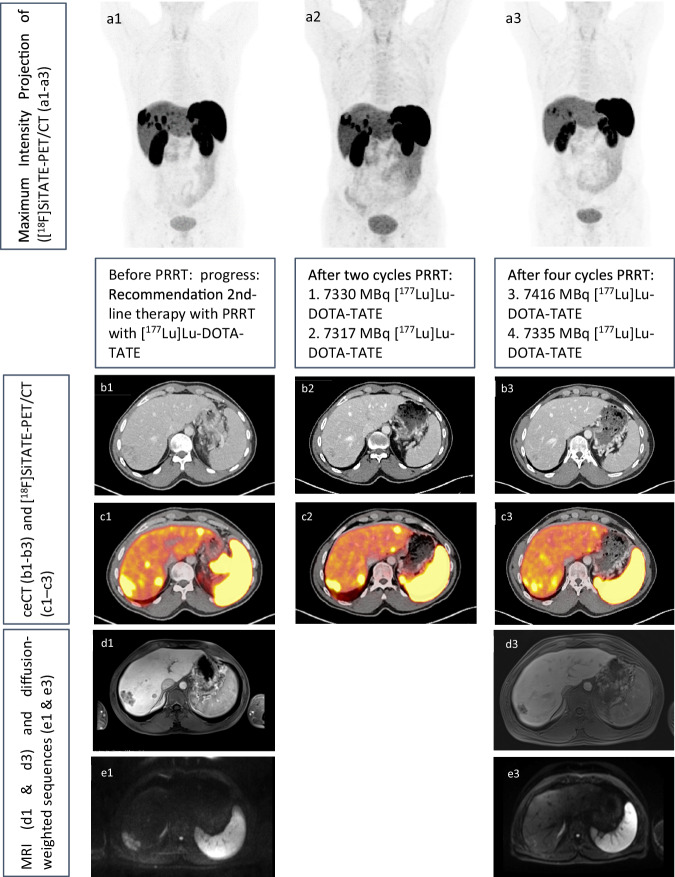
Histologically confirmed NET of the pancreas with multiple liver metastases (G2, Ki67% 10–20%) depicted in [^18^F]-SiFAlin-TATE PET/CT and MRI scan. After chemotherapy with Streptozotocin and 5-Fluorouracil, SSR-PET/CT showed tumor progression, multidisciplinary team recommended second-line PRRT with [^177^Lu]Lu-DOTA-TATE. Comparison of Maximum Intensity Projection (MIP, **a1**–**a3**), contrast-enhanced CT (ceCT, **b1**–**b3**), SSR-PET/CT (**c1**–**c3**), contrast-enhanced MRI (**d1**, **d3**) with diffusion-weighted imaging (DWI, **e1**, **e3**) sequences show a size reduction of the lesions in ceCT and MRI, reduced SSR expression and reduced diffusion restriction as signs of a therapeutic response

The NETTER-1 study has shown good tolerability and efficacy of [^177^Lu]Lu-DOTA-TATE compared to high-dose SSA (“Octreotide”) in patients with progressing, metastasizing midgut NET. Patients with strong SSR expression and stable disease but who have therapy-resistant clinical symptoms are suitable for PRRT [14]. Figure 3 provides a detailed comparison of imaging data from [^18^F]-SiFAlin-TATE PET/CT and MRI scans following second-line PRRT treatment with [^177^Lu]Lu-DOTA-TATE. The figure compares various imaging modalities, demonstrating a reduction in lesion size and SSR expression, along with changes in diffusion characteristics, indicative of a positive therapeutic response.

### [^18^F]FDG-PET/CT

Imaging with the glucose analog [^18^F]FDG is particularly useful in aggressive NEN, such as well-differentiated NET G3 or poorly differentiated NEC, characterized by a high Ki-67 index and typically lower tumor SSR expression. However, [^18^F]FDG-PET/CT can also be useful in NET G1 or G2 to provide prognostic information as high uptake correlates with an increased risk of early progression, while low uptake characterizes rather indolent tumors [19]. Furthermore, [^18^F]FDG-PET/CT can contribute to biopsy planning in cases of unclear disease courses and the characterization of tumor heterogeneity.

Tumor heterogeneity in NEN complicates treatment by creating variability in how different tumor regions respond to therapy. Different parts of the same tumor may show varying levels of receptor expression or metabolic activity, leading to uneven responses to targeted PRRT or chemotherapy. This inconsistency can lead to partial treatment success—some areas of the tumor may shrink or stabilize, while others continue to grow or spread. Additionally, heterogeneity can lead to rapid development of resistance to therapies, as more aggressive tumor clones that are less responsive to standard treatments may emerge. This aspect necessitates frequent reassessment of the disease and may require a combination of therapeutic strategies to manage the disease effectively over time [20].

**Fig. 4:**
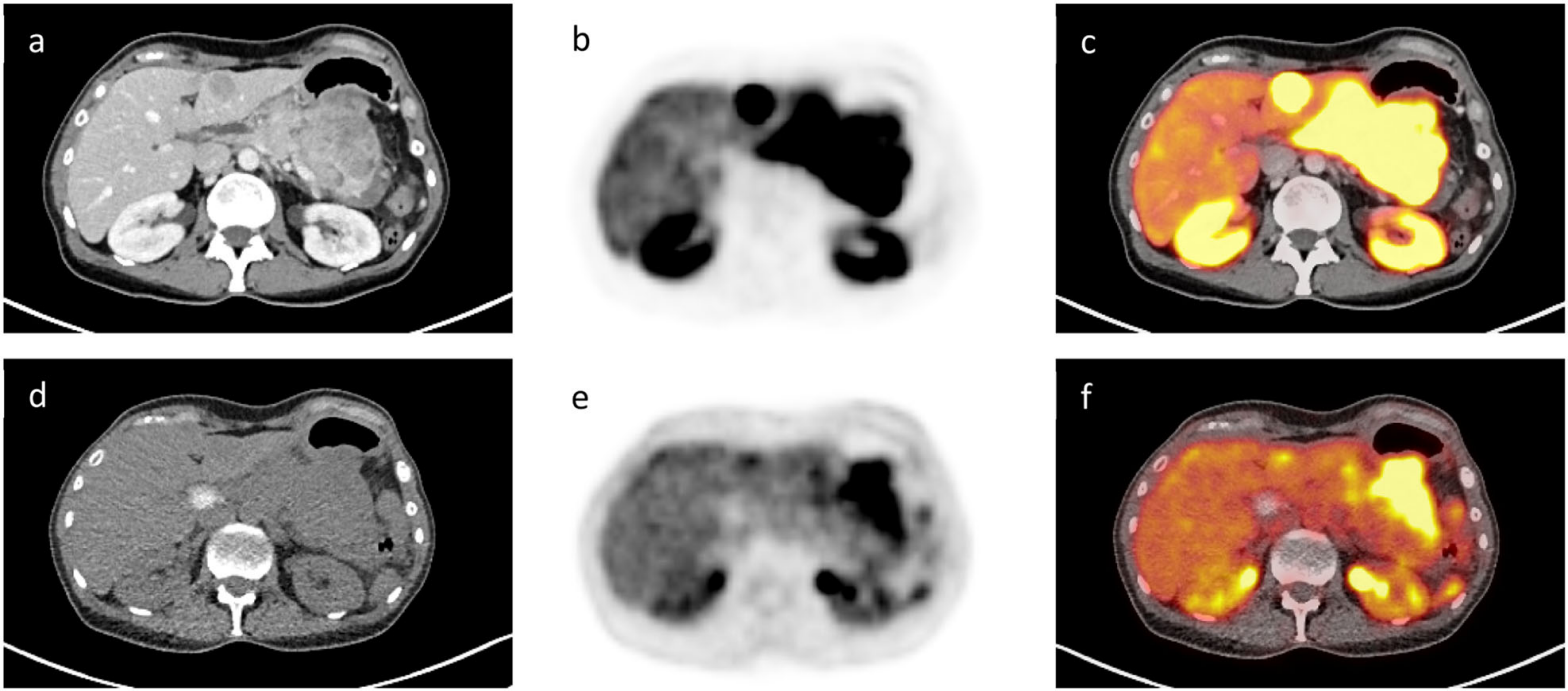
NET of the pancreas, with histological tumor grade G2 and G3. [^18^F]SiTATE PET/CT imaging (top row, **a**–**c**) revealed intense uptake in the body and tail of the pancreas and SSR-expressing liver metastases in segments II/III. FDG imaging (bottom row, **d**–**f**) indicated less extensive metabolic activity in the pancreatic tail, aligning with the histologically confirmed de-differentiated components. The liver metastases exhibited no increased metabolic activity

[^18^F]FDG-PET/CT is also indicated in NET cases where there are suspicious findings on conventional imaging (CT or MRI), but no SSR expression is observed during staging, or in patients with rapid progression of disease despite earlier low-grade disease on pathology. Due to the high risk of metastatic disease, [^18^F]FDG-PET/CT is recommended by the ENETS guidelines for NEC of the gastrointestinal tract before (curative) surgery and adjuvant chemotherapy. Carboplatin combined with etoposide is recommended as the first-line treatment. Should additional treatment be necessary, irinotecan paired with fluoropyrimidines has the best evidence as a second-line treatment [21]. Figure 4 includes comparisons of [^18^F]-SiFAlin-TATE PET/CT and [^18^F]FDG-PET/CT imaging results, illustrating specific uptake patterns and metabolic activity in the pancreas and liver.

## Other imaging modalities in NEN imaging

In patients with NEN, MRI is used in a variety of clinical scenarios. Due to its high soft tissue contrast, it offers high sensitivity for NEN manifestations, particularly of the pancreas, rectum, and liver.

As there is no radiation exposure, MRI is particularly useful for staging and re-staging in younger patients. Some studies have found SSR-PET/CT and whole-body MRI (wbMRI) equivalently accurate in detecting metastases in well-differentiated NET, though they differ in organ-based detection rates [22]. SSR-PET/CT is superior in detecting lymph node and lung metastases, while wbMRI performs better in liver and bone metastases. Overall, SSR-PET/CT is considered superior for NET staging, but individual treatment strategies can benefit from the complementary information provided by SSR-PET/CT and MRI [23].

The integration of DWI sequences into the MRI examination protocol increases diagnostic accuracy, especially for very small liver lesions. Hepatocyte-specific contrast agents also significantly improve sensitivity and specificity for detecting liver metastases—to 91% and 100%, respectively. In comparison with SSR-PET/CT, liver MRI with hepatocyte-specific contrast agent is superior at identifying small liver metastases and should therefore be used routinely before hepatic cytoreduction of NET [24]. MRI of the liver is also recommended when an accurate assessment and precise localization of the tumor burden in the liver are necessary for surgical planning.

Ultrasound plays an important role in the diagnosis and monitoring of NEN. It offers a non-invasive, safe, and cost-effective method, especially for young patients or those with contraindications for CT or MRI examinations. The interpretation of US results depends on the experience and skill of the examining physician. NEN can often be identified with color-coded Doppler sonography due to its frequent hypervascularization. With contrast-enhanced ultrasound (CEUS), early arterial enhancement and venous washout are typical for NEN. CEUS has shown higher diagnostic accuracy in detecting liver metastases compared to conventional sonography [25].

For surgical planning, precise mapping of tumor manifestations in relation to relevant anatomical structures is essential. Therefore, CEUS cannot replace CT, MRI, or SSR/FDG-PET/CT, but is rather used in combination with other imaging methods and laboratory tests to ensure comprehensive diagnosis and treatment planning [26].

While the imaging techniques CT, MRI, and PET/CT provide broad coverage and offer high diagnostic accuracy for primary NEN in various locations, endosonography offers a detailed presentation specifically for small lesions within the pancreas. Endoscopic ultrasound (EUS) represents the most sensitive method for diagnosing pancreatic NEN (pNEN), with a sensitivity of 82–93% and a specificity of 86–95% [27]. EUS has shown significantly higher sensitivity (97%) in identifying pNEN than CT (85%), MRI (70%), and conventional sonography (75.5%) [28]. Furthermore, endosonography enables the performance of fine needle aspiration or biopsies to obtain tissue samples [29].

CT enterography (CTE) and MR enterography (MRE) are advanced imaging techniques that improve the detection of small-bowel NEN. CTE involves image acquisition after the patient ingests neutral or negative oral contrast agents, such as polyethylene glycol, to ensure optimal small-bowel distention. This improves the visualization of the small-bowel wall and allows the identification of small lesions [30]. MRE provides superior soft tissue contrast without using ionizing radiation, applying multiple imaging sequences, including DWI, to provide detailed assessment and evaluate unclear findings [31].

### Follow-up

Follow-up assessments should include monitoring of clinical symptoms, evaluation of biochemical parameters, and both conventional and SSR imaging [8]. The guidelines of the European and American societies regarding the frequency of follow-up imaging for NEN offer varied recommendations, typically suggesting follow-up intervals of 6–12 months in patients with R0/R1-resected NET G1 and NET G2 with low Ki-67 (< 5%), or every 2 to 3 months for tumors with high proliferation rates, such as NET G2 (Ki-67 > 5%) and NEC/NET G3. Similar staging intervals apply to advanced metastatic disease. For patients who have undergone complete tumor resection and are metastasis-free, a follow-up duration of at least 10–15 years is advised, with lifelong monitoring recommended for those with intestinal tumors, although the staging intervals may be extended to 1 to 2 years with increasing length of follow-up (> 5 years), except in NET G3 which requires shorter intervals.

Follow-up is considered unnecessary only in the scenario of an R0-resection of NET G1 of the appendix measuring less than 1 cm, attributed to the minimal risk of metastasis. For an R0-resection of small NET of the rectum, a single endoscopic follow-up examination is sufficient.

The choice of follow-up method is left to clinical discretion and is influenced by factors such as the patient’s age, metastasis pattern, and disease trajectory [32].

### Summary statement

Although CT and MRI are the primary methods for routine oncological staging due to their availability and reproducibility, the integration of nuclear medicine techniques, such as SSR-targeted PET/CT and [^18^F]FDG-PET/CT, represents a significant advancement in the imaging of NEN. PET/CT offers an outstanding insight into the molecular and functional aspects of NEN, enabling superior diagnostic accuracy in localizing primary tumor, accurate staging, and superior assessment of disease progression. Moreover, PET/CT provides critical information for therapeutic decision-making, including the selection of appropriate treatment modalities such as PRRT. The availability of newer radiopharmaceuticals, such as [^18^F]-SiFAlin-TATE, promises further improvements in availability, imaging sensitivity and resolution. Overall, the integration of PET/CT is crucial for the multidisciplinary management of NEN patients, enhancing diagnostic accuracy, treatment efficacy, and prognostic assessment.

### Patient summary

While CT and MRI scans are commonly used because they are widely available and provide consistent results, the integration of advanced nuclear medicine techniques like PET/CT marks a major advancement in the treatment of NEN. PET/CT imaging helps to understand the molecular and functional features of NEN, allowing for the precise localization of primary tumors, accurate evaluation of the spread of the disease, and effective monitoring of progression. They also provide important information that helps doctors choose the best targeted treatments, such as PRRT. This comprehensive integration of PET/CT as a hybrid imaging technique significantly enhances the multidisciplinary management of NEN, supporting diagnostic accuracy and optimizing treatment outcomes.

## Abbreviations

ceCT: Contrast-enhanced computer tomography
CEUS: Contrast-enhanced ultrasound
CT: Computer tomography
CTE: Computer tomography enterography
DWI: Diffusion-weighted imaging
ENETS: European Neuroendocrine Tumor Society
EUS: Endoscopic ultrasound
F: Fluorine
FDG: Fluorodeoxyglucose
G: Grade
Ga: Gallium
Ge: Germanium
GEP: Gastroenteropancreatic
In: Indium
Lu: Lutetium
MRE: Magnet resonance enterography
MRI: Magnetic resonance imaging
NEC: Neuroendocrine carcinoma
NEN: Neuroendocrine neoplasm
NET: Neuroendocrine tumor
PET: Positron emission tomography
pNEN: Pancreatic NEN
PRRT: Peptide receptor radionuclide therapy
RADS: Reporting and Data System
SPECT: Single-photon emission computed tomography
SSA: Somatostatin analog
SSR: Somatostatin receptor
wbMRI: Whole-body magnetic resonance imaging
Y: Yttrium
